# Are retired physicians suitable for the coaching of clerks?

**DOI:** 10.5116/ijme.59bd.5845

**Published:** 2017-09-26

**Authors:** Ivan Bank, Johannes P. de Leeuw, Willem M. Lijfering, Marjolein H.W. de Bois, Theo C.A.M. van Woerkom, Marjo Wijnen-Meijer

**Affiliations:** 1 Leiden University Medical Centre, Department of Education and Training, Leiden, the Netherlands; 2 Alrijne Hospital, Department of Gynaecology, Leiderdorp, Leiden and Alphen aan de Rijn, the Netherlands; 3 Leiden University Medical Centre, Department of Epidemiology, Leiden, the Netherlands; 4 Haaglanden Medical Centre, Department of Rheumatology, Den Haag, the Netherlands; 5 University Medical Centre Utrecht, Centre for Research and Development of Education, Utrecht, the Netherlands

**Keywords:** Coaches, mentorship, tutorship, qualitative study, clerkships

## Abstract

**Objectives:**

To assess whether clerks need coaches, for which issues, and whether retired physicians are able to support clerks.

**Methods:**

The study combines both qualitative and quantitative methods and the perspectives of both coaches and clerks. Clerks starting their first clerkship were randomised between having a coach (n=61) and not having a coach (n=59). All participants were followed for 18 months. Both clerks and coaches completed questionnaires. In addition, in-depth interviews with the coaches were conducted.

**Results:**

Clerks who had a coach were happy to have one. During follow-up, there were no differences between both groups concerning free time, self-esteem, stress, or the number of conflict situations. Clerks with a coach indicated to have less stress as compared to the clerks without a coach (delta values concerning occurrence of stress in clerks with a coach 0.35 [95% confidence interval: -0.07 to 0.77, p=0.10] versus clerks without a coach 0.71 [95% confidence interval: 0.29 to 1.12, p=0.002]). Different issues were discussed with the coaches, such as career issues, deceased patients, unacceptable behaviour of staff, or unpleasant fellow clerks. All coaches liked fulfilling the role of coach. Many found it an interesting way of doing something after their recent retirement from clinical practice. They mentioned that clerks needed them more during the first year of clerkship than later on.

**Conclusions:**

Retired physicians can be used as coaches for clerks. They are well motivated and have enough time for this task. Clerks are enthusiastic about the coaches.

## Introduction

Medical education has become increasingly transformed in recent decades. Students are attending curricula in which they are exposed much earlier to issues concerning daily clinical practice and its many different aspects. Furthermore, as compared to the past, they also attend clinical practice at a much younger age.^1^ This raises the question of whether they are adequately supported. There is a growing number of observations about students’ problems and mental distress.^2^ The various demands of medical education pose a challenge to personal well-being, sometimes even leading to burnout or, for example, erosion of empathy.^3^^-^^6^ It is possible that medical students experience stress due to increased working hours or work load, but it is also possible that they feel uncertain about what is expected of them.^4^^,^^7^ Furthermore, they may not feel comfortable because of their self-perceived lack of knowledge or the fact that they are pushed relatively unprepared to observe medical errors or patients dying.

Research suggests that too many medical students experience burnout. In some studies, a rate almost as high as within the practicing physician population is observed.^8^^,^^9^ Lower support and higher stress appear to be related to burnout. Medical schools offer students help from professionals, like academic counselors, in order to try to help them overcome their problems and to advise them on how to structure or prepare their tasks or exams. These professionals know much about students and their interests in general. They are, however, less experienced in situations associated with daily clinical practice or health care. Therefore, in some countries mentorships are increasingly being developed and introduced.

Mentoring, tutoring, or coaching is mostly defined as ‘a process whereby an experienced, highly regarded, empathic person guides another (usually younger) individual (the mentee) in the development and re-examination of their own ideas, learning, and personal and professional development’.^10^^,^^11^ Although programmes in which mentors support medical students may have pros, they sometimes have limitations as well. For example, many programmes are focused mainly on stimulation of motivation, research productivity, career support, or professional skills.^11^^-^^13^ Personal issues concerning the medical student are not always addressed. Furthermore, it is often challenging to find eligible staff members as mentors who are available and have time to coach or advise students whenever necessary.^14^^,^^15^ Although clinicians are preferred for such a task over non-clinicians, they are often very busy. Moreover, many eligible candidates who could mentor students often have educational tasks, like supervising and assessing clerks. This may interfere with their independence towards help-seeking students. Finally, the situation regarding mentoring programmes in the Netherlands is not well known, because mentoring programmes for medical students in the clinical phase have not yet been studied. Issues occurring during clerkships or what particular help or support should be offered to clerks are not even described.

To fill this gap and to study clerks’ necessities, the present pilot study is designed to assess whether there is a feasible way of supporting clerks by introducing recently retired physicians as their coaches. Accordingly, this study addresses whether these coaches are helpful, and if they are accepted by the clerks. Furthermore, the study considers which issues are addressed and how these coaches deal with their task. To this end, a qualitative study was designed in which perspectives of both coaches and clerks were studied. Clerks starting their first clerkship were randomised between having a coach and not having a coach. Afterwards, all participants as well as participating coaches were followed. Clerks were studied by questionnaires, whereas coaches were studied by questionnaires as well as in-depth interviews.

## Methods

### Study design and participants

This study has both quantitative and qualitative components that were conducted at the medical school of Leiden, the Netherlands. Both clerks and retired physicians who had a role of coach for clerks were studied.

The participating clerks were recruited from the medical school of Leiden. Clerkships start at the beginning of the fifth year of the curriculum and finish during the sixth and final year. Every two weeks a new group of 14 to 16 clerks enrols. All clerks starting between December 2014 and April 2015 were eligible for inclusion in this study. They were approached for participation in the study by the researchers. The study was explained to the clerks in written form as well as orally. If the clerks agreed to participate, written consent was obtained. After that, they received an initial questionnaire. Then, they were randomised either to be assisted by a coach or not. The participants without a coach were considered to be the control group for the group consisting of clerks with a coach. The first follow-up questionnaire was sent to both groups of clerks after eight months, and the second and final one after 18 months. During this follow-up period, the participants with a coach had various group meetings with their coach. Additionally, they could contact their coach themselves whenever they needed to.

The participating coaches were recruited from the area around Leiden. They were all physicians practicing in one of the hospitals working together with the medical school of Leiden, and they needed to be involved in work with clerks in the past. They were all recently retired. Furthermore, it was essential to have excellent references concerning social intercourse with clerks and residents in the past. Other criteria for selection were good communication skills; no involvement whatsoever in medical education for clerks in the present, no known history of indecent situations with students or patients, availability, and sufficient time during the study period.

A list of possible coaches was derived from the hospitals in the area around Leiden. The researchers first inventoried whether these candidates were available for at least 18 months and then asked them if they were interested in participating. After the coaches were selected, they were trained by the researchers on how to respond to possible particular situations. They were told which issues needed to be addressed to professionals within the medical school or hospitals, and how to act in case of doubt. Furthermore, they were offered assistance by an independent physician if they needed advice. This person was a recently retired professor of medicine, not involved as a researcher in this study, and formerly involved in medical education in various roles. Participating coaches received a questionnaire at the start and after 18 months. Additionally, they were interviewed at the end of the study.

After randomisation, the participants randomised for a coach were introduced to their coaches. A total of eight consecutive groups of seven or eight clerks were assigned a coach; the control group included seven or eight clerks who

were not assigned to a coach. The follow-up period was defined as the period between randomisation and the 18th month of clerkship.

Ethical approval was obtained from the university hospital medical ethics committee as well as the medical school and regional hospitals participating in the education of clerks. No risks for students or coaches were determined. All identities of both students and coaches were kept confidential and not mentioned in the transcripts, study reports, or publications related to this study.

### Data collection

In the quantitative study, the data were obtained from both clerks and coaches by using questionnaires. The questionnaire administered at the start of the study was obtained from participants by directly distributing and collecting the paper questionnaires by the researchers. The clerks’ questionnaires after eight months and 18 months were sent by email, whereas the final questionnaire for the coaches was completed on paper directly after the interview.

The questionnaire for the clerks included 30 items. It concerned questions on different issues regarding medical education, private life, and personal coping strategies. The participants who were randomised for a coach were asked also about issues concerning their contact(s) with the coach. Participants who did not respond during follow-up were sent a reminder by email. If a participant declined filling in further questionnaires or was lost to follow-up for other reasons, that participant’s data were included up to the point where participation ceased.

**Table 1:** Participating clerks with a coach and participating clerks without a coach: basic characteristics

Characteristics	Clerks with a coach	Clerks without a coach	Total
	Mean (SD)	mean (SD)	Mean (SD)
Age, years	24 (1.72)	24 (1.36)	24 (1.55)
Feels happy	4.00 (0.94)	4.07 (0.83)	4.04 (0.88)
Satisfied with his/her level of knowledge	3.39 (0.74)	3.19 (0.66)	3.30 (0.70)
Considers himself/herself to be a good student	4.02 (0.67)	4.01 (0.54)	4.02 (0.61)
Considers his/her private situation to be stable	4.03 (1.01)	4.20 (0.89)	4.12 (0.95)
Busy social life	3.75 (0.83)	3.94 (0.86)	3.85 (0.85)
Has much stress	2.43 (0.96)	2.36 (1.04)	2.40 (0.99)
Used to discusses medical school issues with			
Classmates	3.95 (0.83)	4.14 (0.91)	4.05 (0.87)

The questionnaire for the coaches included 20 items. Topics of this questionnaire were experiences with the group, issues that were discussed, number of meetings, time spent with clerks, and whether they themselves were satisfied with the results of their interventions or support. Both questionnaires had never been validated.

In the qualitative study, topics were generated a priori on the basis of hypotheses and study of the scarce literature concerning coaching of students and trainees. The in-depth interviews with the coaches were semi-structured and contained the following topics: How was your first impression of the group and how was the general atmosphere? What issues were usually discussed? Did really serious problems occur? How much time did you spend coaching? Workload? Generational gap? Did you find yourself to be capable of dealing with the issues/problems? How was this period overall? Any suggestions for colleagues wanting to become a coach for clerks too?

All eight participating coaches were interviewed by IB, a physician experienced in in-depth interviewing. One interview took place at the participant’s home, whereas the others were interviewed in the Medical School building of the University of Leiden. All interviews were tape-recorded with consent and transcribed later.

### Data analysis

The data from the questionnaires were analysed using the SPSS 23 programme. We summarised personal and baseline data as means with standard deviations or proportions. We used Student’s t-test among groups with and without a coach for comparing distribution of the mean differences between persons. With a paired t-test, we compared the difference between the mean changes in baseline and follow-up data within persons. In this pilot study, it was decided a priori not to perform a power calculation but to use a sample size that was manageable for the purposes detailed in this paper.

Concerning the interviews, three investigators (IB, JPL, MWM) carried out the analysis independently by the established rules of performing qualitative data analysis.^16^ Their independent results were compared, and central themes were used for further analysis. Data processing and analysis was an ongoing process in which central questions were identified through a preliminary analysis.16 Later on, data from different interviews were categorised by these themes and compared by constant comparison. Differences were discussed until consensus was reached.

## Results

### Clerks

In total, 124 medical students started clerkships during the period of inclusion. Of them, 120 clerks consented to participate in the study. Sixty-one participants were randomised to have a coach, and 59 participants formed the control group. As may be expected based on the student population, about 63% of the participating students were female. Table 1 shows that there were no significant differences between the two groups concerning basic characteristics. Most of the participants felt happy with their life and were satisfied with their functioning as medical students.

The final follow-up questionnaire after 18 months was completed by 68 participants (57%; of them, 34 participants were randomised to have a coach).

**Table 2:** Participating clerks with a coach and participating clerks without a coach: 18 months’ follow-up during clerkships

Item	Clerks with a coach	Clerks without a coach	Mean difference (95% confidence interval)	p
Considers himself/herself to be a good student	4.29	4.38	-0.09 (-0.46 to 0.28)	0.64
Successful clerkships	4.32	4.38	-0.06 (-0.40 to 0.28)	0.74
Satisfied with his/her level of knowledge	3.71	3.53	0.21 (-0.24 to 0.60)	0.40
Feels happy	3.76	3.79	-0.02 (-0.55 to 0.50)	0.91
Finds clerkships to be hard	3.02	2.94	0.09 (-0.47 to 0.64)	0.75
Clerkships are just as expected	3.74	3.88	-0.15 (-0.57 to 0.28)	0.49
Need to talk to someone	3.55	3.20	0.35 (-0.24 to 0.95)	0.24
Has experienced a conflict	2.61	2.35	0.26 (-0.39 to 0.92)	0.43
Has experienced more than one conflict	1.82	1.76	0.06 (-0.45 to 0.57)	0.82
Has much stress	2.85	2.94	-0.09 (-0.64 to 0.46)	0.75
Considers his/her private situation to be stable	3.97	4.00	-0.03 (-0.58 to 0.52)	0.92
Busy life	3.76	3.67	0.09 (-0.36 to 0.53)	0.69
Feels tired all the time	2.82	2.79	0.03 (-0.59 to 0.65)	0.93
Enough time for hobbies or sports	3.52	3.38	0.15 (-0.43 to 0.73)	0.61

 “We have a nice and empathetic coach, and the meetings with the group are always pleasant.” (participant 31, female)

Different issues were discussed in the group meetings as well as between coaches and individual clerks. These issues are listed in Table 2. In the group sessions, most often the clerks’ daily work in the hospital, the difficulties they had with some physicians or fellow clerks, and important patient issues were discussed. These issues concerned end-of-life topics, difficult clinical entities, as well as their feelings about talking about or observing patient-related problems (e.g. smells, faeces, rectal or vaginal examinations). Furthermore, many clerks took the opportunity to talk about possible careers, research possibilities, networking, and differences between hospitals and centres in the Netherlands.

One of the participants stated it like this:

“We were talking about how to choose specialisations, about how to manage our work with unpleasant staff members. The dinners were pleasant, and it was interesting to hear how others think about particular issues or how they manage them.” (participant 73, male)

The issues discussed between coaches and individual clerks concerned different subjects, like the intention of quitting clerkships, an unexpected difficult start of a clerkship, or, in one case, unacceptable behaviour of a hospital staff member. Statistical analysis showed that there were almost no differences between both groups concerning daily life, free time, self-esteem, stress, or the number of conflict situations during the follow-up (Table 2). However, clerks who were assigned a coach generally felt better and happier than clerks without a coach (Table 3). Furthermore, clerks with a coach indicated to have less stress as compared to their controls at the final measurement. Sub-analyses did not show differences between coaching groups themselves (data not shown).

Many participants stated not to have needed a coach at all, although most of them were happy that a coach was available in case they get into situations in which they need a coach. Some illustrated it like the following three participants:

“I did not need him myself. Nevertheless, I think that it might be useful for the ones who do not have people around to talk to, or the ones involved in more conflicting situations.” (participant 7, male)

“I needed him once - it was a good thing! I do not need him now anymore.” (participant 19, female)

“I consider it useful to have someone of trust not involved in the hospital or medical school.” (participant 109, female)

None of the participants suffered disease needing hospital treatment or quit a clerkship. However, in one case a clerk was advised by his coach to visit his general practitioner for further help. It concerned symptoms due to a psychiatric disorder.

### Coaches

All coaches were enthusiastic about the pilot study and felt proud that they were asked for this task. Many of them found it an interesting way of doing something after their recent official retirement from clinical practice. They were available most of the time for the clerks. All of them said that they had no time issues and that this project did not consume too much time or energy.

**Table 3:** Differences between both groups of clerks during follow-up

Status	Clerks with a coach	Clerks without a coach	Mean difference of the difference (95% confidence interval)
Feels happy	-0.29 (-0.65 to 0.07)* p=0.11	-0.39 (-0.74 to -0.03)* p=0.04	0.09 (-0.40 to 0.58) p=0.72
Successful clerkships	0.26 (0.17 to 0.51)* p=0.04	0.26 (-0.05 to 0.57)* p=0.10	0.00 (-0.39 to 0.39) p=1.00
Occurrence of stress	0.35 (-0.07 to 0.77)* p=0.10	0.71 (0.29 to 1.12)* p=0.002	-0.35 (-0.93 to 0.23) p=0.23

During the follow-up, all coaches had meetings with members of their group on a regular basis, except for one coach. This coach failed in his attempts to make an appointment with the group members after the first meeting. The students of this group also did not contact their coach themselves. Three of the other coaches had two meetings with their groups; one coach had three meetings; one coach had four meetings; and two coaches had more than four meetings. Additionally, the coaches had a varied amount of contact moments with individual group members. They liked to spend time with the clerks and did not think the generation gap was a problem in the contacts with the clerks. They were all of opinion that persons with a history of good relationships with clerks and residents were capable of taking on this duty too.

For a good relationship between the coach and the group, sufficient coherence among the individual students within the group is needed.

“They did not become part of a group. No. They really did not become part of a group.” (coach 3)

The coaches who had the most frequent group meetings all said that it was mostly due to the group characteristics and an easy start during the introduction meeting. According to the coaches, mentorship is a relationship rather than just a set of activities. All stated that it is a developmental process for both parties. Some said they thought that their success was mostly due to the fact that they were listeners rather than managers. Trust, however, is even more important, in their opinion:

“It is important to be trusted…. Trust needs to develop. How do I become trusted? I need to talk, to talk also about myself and some of the difficulties I had experienced myself, including the fact that I also made mistakes by, for instance, operating on the wrong knee….” (coach 1)

One of the coaches stated that the independence of the university clinic or hospital appears to be of importance too. For example, in one case some issues with a well-known staff member could be discussed by the group without anxiety among the students that this would have an impact on their final marks. This may be illustrated by the following quote:

“It was really difficult for them to give feedback to the staff member, both written and verbal, because they were afraid that it would influence their final mark….” (coach 5)

The coaches felt that the clerks needed them more often during the first year of clerkship as compared to the second year. The issues most discussed are listed in Appendix 1.

All coaches wanted to see or help the clerks more than they were asked, and some of them were a little disappointed that it was difficult to make new appointments or that, in some cases, the students stopped coming or contacting. Sometimes it concerned logistical reasons. Clerks work in different hospitals in the region, and it is not easy to find a time or location that fits all clerks. Nevertheless, motivational issues might also play a role, and a more casual coaching programme is suggested by some coaches.

None of the coaches needed support from the independent physician. All of them told the interviewer spontaneously that they wanted to be asked to participate again if this pilot study would turn into an official programme. They also described how a coach should be:

“You need to focus on how it is done now, in the present.” (coach 7)

“I would state: be a mate, not the old-fashioned physician; be flexible; be interested in issues of this present generation.” (coach 7)

None of the students appeared to have questions concerning drug or alcohol abuse that he or she wanted to discuss with the coach. There were also no issues concerning pregnancy, financial problems, or bad marks discussed with the coaches. Nevertheless, in rare cases a coach was asked for advice about a private problem, for example, an unstable situation at home.

## Discussion

The data reported in this study show that clerks are happy to have a coach, especially when they need one, and that these coaches may be selected amongst retired physicians with good communication and social skills. Potentially, the group of experienced and motivated retired physicians with a clinical background is an inexhaustible source.

First, we found that coached clerks rate their overall well-being as higher compared to non-coached clerks. They also appear to have less stress in comparison to their controls. This is in accordance with other studies, such as that of Macaulay and others.^17^^,^^18^ Despite the presence of a coach, this effect may also have been induced by peer support during the group meetings, because students are known to often receive advice from their peers. However, it is also known that students seeking help do not always follow the advice given by their fellow students.^19^ Fellow students often lack the experience or seniority. Nevertheless, next to these possible mechanisms, it also needs to be studied whether these positive effects also would influence the occurrence of burnout amongst clerks. However, the students find it useful to have available an independent coach with knowledge about daily clinical practice and the world of health care, and possibly this feeling of security is just enough to help them carry on. On the other hand, it is known from the literature that dysfunctional mentoring relationships may have a negative impact on individuals.^20^ This may be overcome by offering personal matching between students and mentors, although that is a very time-consuming and costly intervention.^21^ It is possible to offer a less rigid mentoring programme as we did, and to let the students choose for themselves if they need a coach and/or when they need him or her. However, if it is too casual, some students might not contact their coach at all, as happened in one of the studied groups. A successful mentor–mentee relationship requires the active participation of both parties. For the coaches’ point of view, forming a relationship is essential, so it is a relationship rather than just a set of activities, and it should be a developmental process for both parties.

According to Frei et al., an effective mentor should be available, non-judgmental, able to encourage the mentee, be a role model, build a professional network, and assist in the mentee’s personal development.^11^ On the contrary, a main pitfall of mentoring is time pressure as well as the fact that many mentors offer solutions instead of enabling students to find their own solutions and support that process.^11^^,^^22^ Last but not least, it is well known that it is hard to find mentors.^23^ We are of the opinion that retired physicians might fulfil all of these requirements and that they are more available. And, although there is still no data available on cost-effectiveness, the coaches we would propose are less costly. Whether financial compensation should be offered still needs to be discussed. Furthermore, age and duration of retirement are also factors of importance to be discussed. In all cases, the coaches should be absent too long from clinical practice, although it is difficult to give an exact limit, especially because everyone is different. Coaches should be asked and evaluated after some time.

Finally, it is interesting to find out what kinds of problems are discussed. For anyone working in health care, some issues are obvious, like the fact that students need to talk about difficult patients or conflicts. Nevertheless, it was not well known for the Dutch population of clerks, and we found out that they also needed to ask an experienced physician how to talk about ‘filthy issues’. It appears that many do not ask this of their official supervisors, and we do not know if it concerns taboos or that they do not want the staff members to know that they do not know or feel comfortable about these things. Furthermore, issues of inappropriate behaviour as well as unpleasant colleague-clerks were discussed.

In our view, a feasible model for a student mentoring programme could be designed by involving enthusiastic retired clinicians. Instead of randomly forming groups of clerks, the groups should be formed with clerks working in the same hospitals, making meetings easier to organise. An optimal group consists of seven or eight members. A coach needs time to build a tight relationship with the clerks. In all situations, the clerks should be in charge and ask about their needs. The coach should be available for both the group and one-to-one contacts.

Our study has some limitations. First, our final follow-up questionnaire response rate was 57%. Because of our study design, we did not want to pressure the participants too much by asking them to complete the questionnaire. As investigators not involved in the intervention, we wanted them to feel free and safe and not to interfere with the coaching or the groups. However, the responses were the same from both the coached participants and the control group. Therefore, we consider it unlikely that the response rate of 57% led to significant bias. Furthermore, most of the quantitative analyses were not sufficiently powered to find statistically significant findings. For this reason, results from the quantitative analyses must be handled with caution. Then, participants were randomly assigned to coaches, although consecutively. From the literature, it is known that randomly assigning protégés to mentors might lead to disappointing results.^21^^,^^24^ We do not know if that happened in our study, affecting our results. Nevertheless, we do not have one single observation from students that they disliked their coach and that that was the reason they did not want to contact their coach any longer or get together. Then, our questionnaires had never been validated. While designing our protocol, we were of the opinion that this concerned a pilot study focusing on qualitative analyses, and a reliability analysis was never conducted. Finally, concerning the questionnaire provided to the coaches, the answers to the questions in the second questionnaire may have been influenced by the interviews. On the day of the interview the participating coaches were interviewed first, and then they answered the questionnaire.

## Conclusions

The findings of this first Dutch study on mentoring clerks show that clerks sometimes need a coach and that they are happy to have one. Furthermore, coaching is associated with positive outcomes concerning the occurrence of stress and the overall well-being of clerks. Many different issues are discussed within group meetings as well as in one-to-one contacts. Coaches may be selected amongst retired physicians with good communication and social skills. They are highly motivated for this task and do not find it difficult or time-consuming.

Both medical schools and clerks may benefit from the observations of this study. Concerning the medical schools, this study shows a feasible and profitable solution for the relative shortage of available clinicians for tutoring during clerkships. Concerning medical students and medical education in general, potential deployment of retired medical professionals may offer additional help and attention to clerks. This attention is needed and asked for, so it needs to be offered to clerks, at least in the Netherlands.

### Acknowledgements

We wish to thank all eight coaches for their participation as well as for their great enthusiasm. We also thank professor Jary van Baalen for his help and his role as independent advisor for the coaches.

### Conflict of Interest

The authors declare that they have no conflict of interest.

## Supplementary material

Supplementary file 1 Appendix 1. Issues discussed between clerks and their coaches
