# Supplementary material for: Are retired physicians suitable for the coaching of clerks?
Source: Int J Med Educ. 2017 Sep 26;8:343–50. doi: 10.5116/ijme.59bd.5845 (PMC5694694; doi:10.5116/ijme.59bd.5845)
Supplement: Supplementary file 1 — Appendix 1. Issues discussed between clerks and their coaches [file ijme-8-343-S1.pdf]

**Appendix 1**
**Issues discussed between clerks and their coaches**
Career
Difficult clinical entities
Deceased patients
Feeling that a staff member does not likes him or her
Difficulties performing rectal or vaginal examinations
Difficult to discuss or foul patients' characteristics
Help finding an interesting clerkship abroad
Unacceptable behaviour of staff members
Conflicts between physicians
Unpleasant clerks
Optimal behaviour during clerkships
Quitting clerkship
